# Psychological factors underpinning vaccine willingness in Israel, Japan and Hungary

**DOI:** 10.1038/s41598-021-03986-2

**Published:** 2022-01-10

**Authors:** Robin Goodwin, Menachem Ben-Ezra, Masahito Takahashi, Lan-Anh Nguyen Luu, Krisztina Borsfay, Mónika Kovács, Wai Kai Hou, Yaira Hamama-Raz, Yafit Levin

**Affiliations:** 1 Warwick University, Psychology, Coventry, CV4 7AR UK; 2 Present Address: Ariel University, Social Work, Ariel, Israel; 3 Yamaguchi University, Humanities, Yamaguchi, Japan; 4 Institute of Intercultural Psychology and Education, Eötvös Loránd University, Budapest, Hungary; 5 Education University Hong Kong, Psychology, Ting Kok, Hong Kong; 6 University of Zurich, Psychology, Zurich, Switzerland

**Keywords:** Human behaviour, Infectious diseases, Viral infection

## Abstract

The spread of SARS-CoV-2 led to rapid vaccine development. However, there remains considerable vaccine hesitancy in some countries. We investigate vaccine willingness in three nations with very different vaccine histories: Israel, Japan and Hungary. Employing an ecological-systems approach we analyse associations between health status, individual cognitions, norms, trust in government, COVID-19 myths and willingness to be vaccinated, with data from three nationally representative samples (Israel, Jan. 2021, N = 1011; Japan, Feb. 2021, N = 997; Hungary, April 2021, N = 1130). Vaccine willingness was higher in Israel (74%) than Japan (51%) or Hungary (31%). In all three countries vaccine willingness was greatest amongst who would regret not being vaccinated and respondents who trusted their government. Multi-group latent class analysis identified three groups of COVID myths, with particular concern about alteration of DNA (Israel), allergies (Hungary) and infection from the vaccine (Japan). Intervention campaigns should address such cultural myths while emphasising both individual and social benefits of vaccination.

## Introduction

As the world-wide threat posed by the SARS-CoV-2 virus continues the development and implementation of vaccines has become pivotal for reducing mortality and morbidity and limiting spread^1^. However, the availability of vaccines, speed of vaccination and willingness to vaccinate varies substantially across cultures and is affected by historical and political factors. Three countries exemplify such influences. Israel was the first country to launch a mass vaccination drive. Starting on 20th December 2020, 15% of the country’s population had received at least their first (Pfizer-BioNTech) vaccination within two weeks, 39% within a month^2^. Comparative surveys (late January 2021) found 73% of Israelis willing to accept a vaccine^3^. In Japan, problems with different vaccines during the 1970s and 1980s, and wide-spread concern about HPV vaccination in 2013, led to a risk-averse approach^4,5^, with Japanese regulators not approving a COVID-19 vaccine until mid-February 2021^2^. Only 45% of the Japanese surveyed in late January 2021 indicated willingness to take an approved COVID-19 vaccine^3^. Finally, in Hungary, a limitation in supply of vaccines (in particular Pfizer-BioNTech) contributed to slow initial uptake of a COVID-19 vaccine^2,6^. Surveys in Hungary suggested only 38%^7^–45%^8^ were willing to vaccinate during the autumn of 2020, although later studies suggested some gradual increase in vaccine willingness^8^.

**Figure 1:**
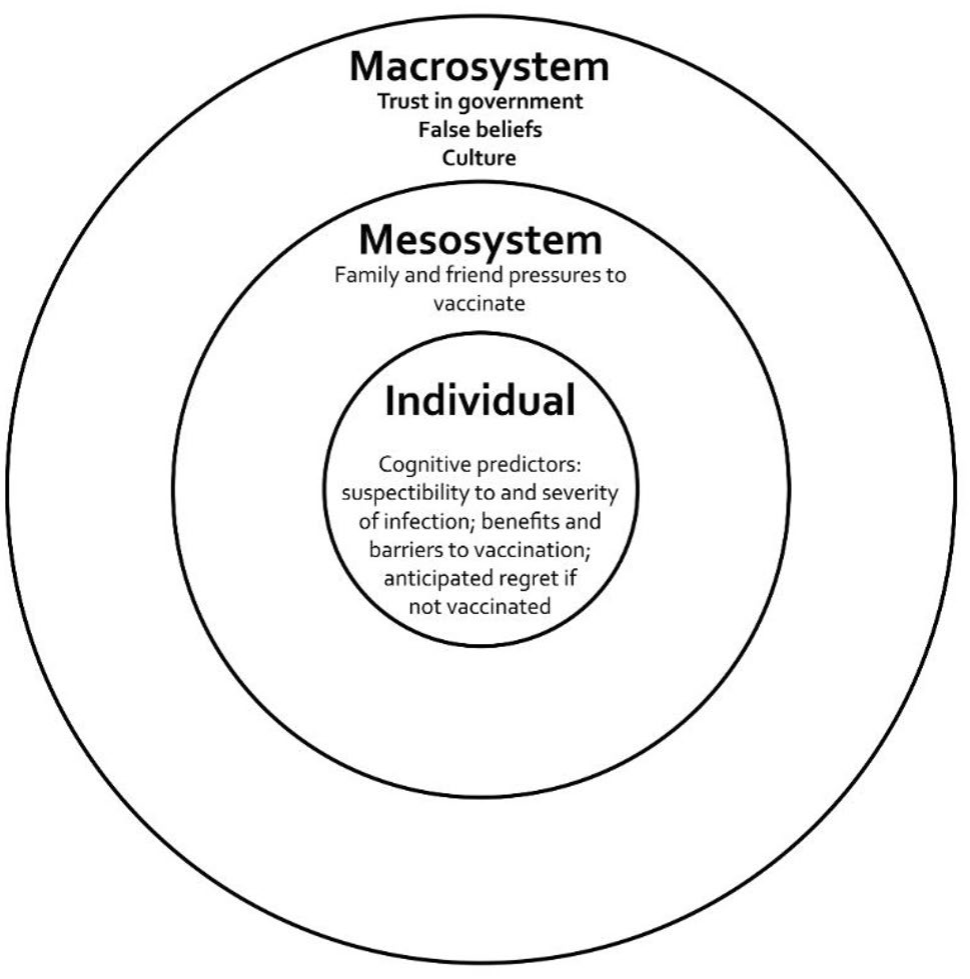
Ecological model of factors contributing to vaccine willingness.

Alongside such historical-cultural factors vaccination willingness also varies by both demographic and individual, psychological differences within countries. In Israel, some religious communities were more likely to reject the vaccine^9,10^. Vaccine willingness was greater in Japan amongst men, older populations and those with chronic disease risk factors^11^. In Hungary the more educated were more willing to vaccinate^8^. Several individual socio-psychological variables also influence vaccine uptake^12–14^, although a paucity of theory-driven approaches to vaccination means there are a limited number of systematic frameworks available^15^. In this paper we draw on the three most widely employed psychological models of vaccine willingness—the Health Belief Model, the Theory of Planned Behaviour and Protection Motivation Theory^14,16^—to suggest an ecological systems model^17^, organised into three nested categories, capturing micro, meso and macro-system influences on vaccine willingness (Fig. 1). The model includes (1) individual cognitions involved in decision-making (perceptions of susceptibility and severity of illness, perceived benefits or barriers to vaccination, and anticipated regret if not vaccinated) (2) local group influences and norms (the influence with important others, including family and friends) and (3) wider macro cultural factors, including communication, trust in government and health authorities. We assess this ecological model during the very different vaccination drives in Israel, Japan and Hungary. Vaccine willingness is anticipated to be positively related to both threat appraisal and the ability to confront this; specifically, perceived susceptibility of contracting COVID-19, severity of COVID-19, benefits of vaccination, and anticipated regret if not vaccinated^11,14^. Normative pressures to vaccinate are also anticipated to encourage willingness to vaccinate, while low trust, and a willingness to accept misbeliefs about vaccination, are expected to be negatively associated with willingness to vaccinate^14^. We control in these analyses for the key demographic variables of age (positively associated with vaccine willingness^12,14^, education (positively associated with uptake^13^) and sex (with men more willing to vaccine^11,12^). In Israel only we also include religiosity, anticipated to be negatively associated with vaccine willingness^12^. Trust in government authorities is often negatively associated with conspiracy or false beliefs about a pandemic^18–20^. Misbeliefs, which often focus on the ‘response cost’ of vaccinations^21^, are particularly significant given the novelty of the vaccines developed^18,22^, with vaccine side-effects the most prominent concern^20^. While these misbeliefs tend to correlate significantly with each other^19,20^, they may also be divided along dimensions^18^. In addition to testing our ecological model we examine the clustering of these myths in each country via multi-group latent class analysis (LCA), associating each class in each country with willingness to vaccinate.

This paper seeks to address three objectives. First, we compare rates of vaccine willingness across Israel, Japan and Hungary (**Objective 1**). Second, we examine the relative weight of each of the predictor variables in our three-layer ecological model by conducting a multigroup path analysis in each culture (**Objective 2**). Finally, (**Objective 3**), we conduct a sensitivity analysis examining specific cluster structure of myths about COVID-19, and the impact of these on willingness to vaccinate in each country.

## Results

### Objective 1: vaccine willingness in Israel, Japan and Hungary

Table 1 presents differences in the distribution of background and study variables between the three samples. Willingness to vaccinate was higher in Israel (74.1%) than in Japan (51.1%), or Hungary (31%) (χ^2^ (2) = 397.86, P = 0.001).

**Table 1:** Sociodemographic characteristics and variables assessed.

Variable [scale reliabilities, *r*.]	Frequency N (%)			Mean (SD)			Country comparison	p
	Israel	Japan	Hungary	Israel	Japan	Hungary		
**Accept the vaccine**								
Disagree	261 (25.8)	448 (48.9)	780 (69.0)				χ^2^ (2) = 397.86***	< .001
Agree	750 (74.1)	469 (51.1)	351 (31)					
Age				39.95 (14.15)	45.63 (14.11)	50.49 (15.95)	F (2, 3124) = 134.92	< .001
Sex (female)	514 (51)	514 (52)	510 (45)				χ^2^ (2) = 10.45*	.032
**Risk group**								
Yes	248 (24.5)	190 (19.1)	469 (41.5)				χ^2^ (4) = 274.803***	< .001
No	763 (75.5)	804 (80.9)	602 (53.2)					
**Education**								
Non academic	580 (57.4)	506 (50.8)	691 (61.1)				χ^2^ (1) = 54.23***	< .001
Academic	431 (42.6)	473 (47.4)	439 (38.8)					
Diagnosed with COVID-19 (yes)	63 (6.2)	2 (0.2)	124 (11.1)				χ^2^ (1) = 110.44***	< .001
Family with COVID-19 (yes)	377 (37.3)	11 (1.1)	687 (60.7)				χ^2^ (1) = 841.70***	< .001
Self-rated health (4-point scale)				3.19 (.60)	2.30 (.78)	2.71 (.77)	F (2,3136) = 374.92*** ŋ^2^ = 0.20	< .001
Likelihood of infection [.73, .68 .85]				2.69 (.89)	2.64 (.72)	2.79 (1.04)	F (2, 3115) = 8.09*** ŋ^2^ = 0.01	< .001
Perceived severity of infection [.77, .67, .80]				3.18 (.98)	3.44 (.73)	3.34 (1.01)	F (2, 3115) = 19.20*** ŋ^2^ = 0.01	< .001
Benefits of vaccination [.79, .74, .91]				3.72 (.99)	3.53 (.78)	3.39 (1.22)	F (2, 3056) = 28.71*** ŋ^2^ = 0.02	< .001
Barriers to vaccination [*r* = *.33, .47, .21*]				3.87 (1.84)	2.95 (.91)	2.44 (.99)	F (2, 3056) = 320.16*** ŋ^2^ = 0.17	< .001
Anticipated regret				4.95 (1.97)	4.41 (1.46)	4.35 (2.14)	F (2, 3061) = 30.95*** ŋ^2^ = 0.02	< .001
Subjective norms [.64, .45, .66]				4.89 (1.08)	4.50 (.82)	4.23 (1.32)	F (2, 3080) = 94.84*** ŋ^2^ = 0.06	< .001
Trust government [.82, .87, .95]				2.74 (1.07)	2.57 (.83)	2.41 (1.28)	F (2, 3087) = 24.56*** ŋ^2^ = 0.02	< .001
COVID-19 myths 1(no) 2 (yes)				1.22 (.24)	1.28 (.21)	1.25 (.24)	F (2, 3127) = 22.98*** ŋ^2^ = 0.01	< .001

***p < .001 ** p < .01 *p < .05.

### Objective 2: testing the ecological model

In **Israel (**Table 2), men were more willing to vaccinate (β = 0.05, p = 0.018). Those with higher subjective rated health were less willing to vaccinate (β = −0.05, p = 0.045). There were positive associations between willingness to vaccinate and the cognitive factors of *benefits of vaccine* (β = 0.17, p < 0.001), *anticipated regret* (β = 0.28, p < 0.001), and *subjective* norms (β = 0.08, p < 0.003) while *barriers to vaccination* were associated with reluctance to vaccinate (β = −0.13 p < 0.001). *Trust in government* was positively associated with willingness to vaccinate (β = 0.06, p = 0.012), and *false beliefs about COVID-19* significantly associated with unwillingness to vaccinate (β = −0.16, p < 0.001). In **Japan,** education was positively associated with willingness to vaccinate (β = 0.06, p = 0.036). There were positive associations between willingness to vaccinate and the cognitive factor of *anticipated regret* if not vaccinated (β = 0.16, p < 0.001) while *barriers to vaccination* were associated with reluctance to vaccinate (β = −0.40 p < 0.001). *Subjective norms* were associated with willingness to vaccinate (β = 0.11 p = 0.001) as was *trust in government* (β = 0.13, p < 0.001). Finally, in **Hungary,** men were more willing to vaccinate (β = 0.11, p < 0.001), and there were positive associations between willingness to vaccinate and *benefits of vaccine* (β = 0.22, p < 0.001), *anticipated regret* (β = 0.22, P < 0.001) and *trust in government* (β = 0.37, p < 0.001). The variances explained were 44.2%, 47.9% and 39.2% in Israel, Japan and Hungary, respectively.

**Table 2:** Magnitude, statistical significance and odds-ratio for willingness to vaccinate.

	Israel (N = 1,011)					Japan (N = 917)					Hungary (N = 1130)				
	b	SE	Est\Se	P	OR	b	SE	Est\Se	P	OR	b	SE	Est\Se	P	OR
Sex (1 = men)	.05*	.02	2.36	.018	1.25	.02	.03	.66	.512	.73	.10***	.02	4.36	< .001	1.99
Age	.00	.00	−.10	.923	.99	.00	.00	.04	.965	1.00	.00	.00	−.22	.824	1.00
Education (1 = academic)	−.01	.02	−.64	.523	.98	.06*	.03	2.09	.036	1.12	−.02	.03	−.76	.450	.98
SRH (4 = excellent)	−.04*	.02	−2.01	.045	.66	.01	.02	.39	.699	1.01	.01	.02	.84	.399	1.01
Risk (1 = risk group)	−.01	.02	−.33	.740	.98	−.03	.03	−.93	.354	.67	−.03	.02	−1.24	.214	.87
Had Covid (1 = yes)	−.03	.04	−.64	.520	1.34	−.04	.06	−.73	.465	.98	−.00	.04	−.09	.926	1.01
Family Had (1 = yes)	.00	.02	−.33	.744	.97	.00	.07	.02	.987	1.00	−.01	.02	−.45	.651	1.05
Perceived likelihood	.02	.01	1.45	.146	1.01	−.03	.02	−1.62	.105	.82	−.01	.02	−.77	.439	.94
Perceived severity	−.01	.01	−.66	.509	.97	−.01	.02	−.40	.688	1.05	−.02	.02	−1.35	.178	.84
Benefit of vaccine	.08***	.02	5.36	< .001	2.21	.04	.02	1.66	.096	1.15	.08***	.02	4.88	< .001	1.92
Barriers to vaccine	−.06***	.01	−4.65	< .001	.72	−.23***	.02	−10.70	< .001	.20	.00	.01	.11	.909	1.02
Anticipated Regret	.07***	.01	8.49	< .001	1.74	.05***	.01	3.90	< .001	1.74	.05***	.01	5.25	< .001	1.37
Subjective norms	.03**	.01	2.96	.003	1.56	.07*	.02	3.40	.001	1.45	−.01	.01	−.46	.646	1.03
Trust in Government	.03*	.01	2.52	.012	1.53	.08***	.02	4.50	< .001	1.98	.13***	.01	12.50	< .001	2.20
COVID-19 myths	−.33***	.07	−4.99	< .001	.11	−.00	.09	−.04	.968	.99	.01	.06	.11	.909	.66

SRH = subjective rated health. ***p < .001 **p < .01 *p < .05.

### Objective 3: Clustering of beliefs across samples

**Figure 2:**
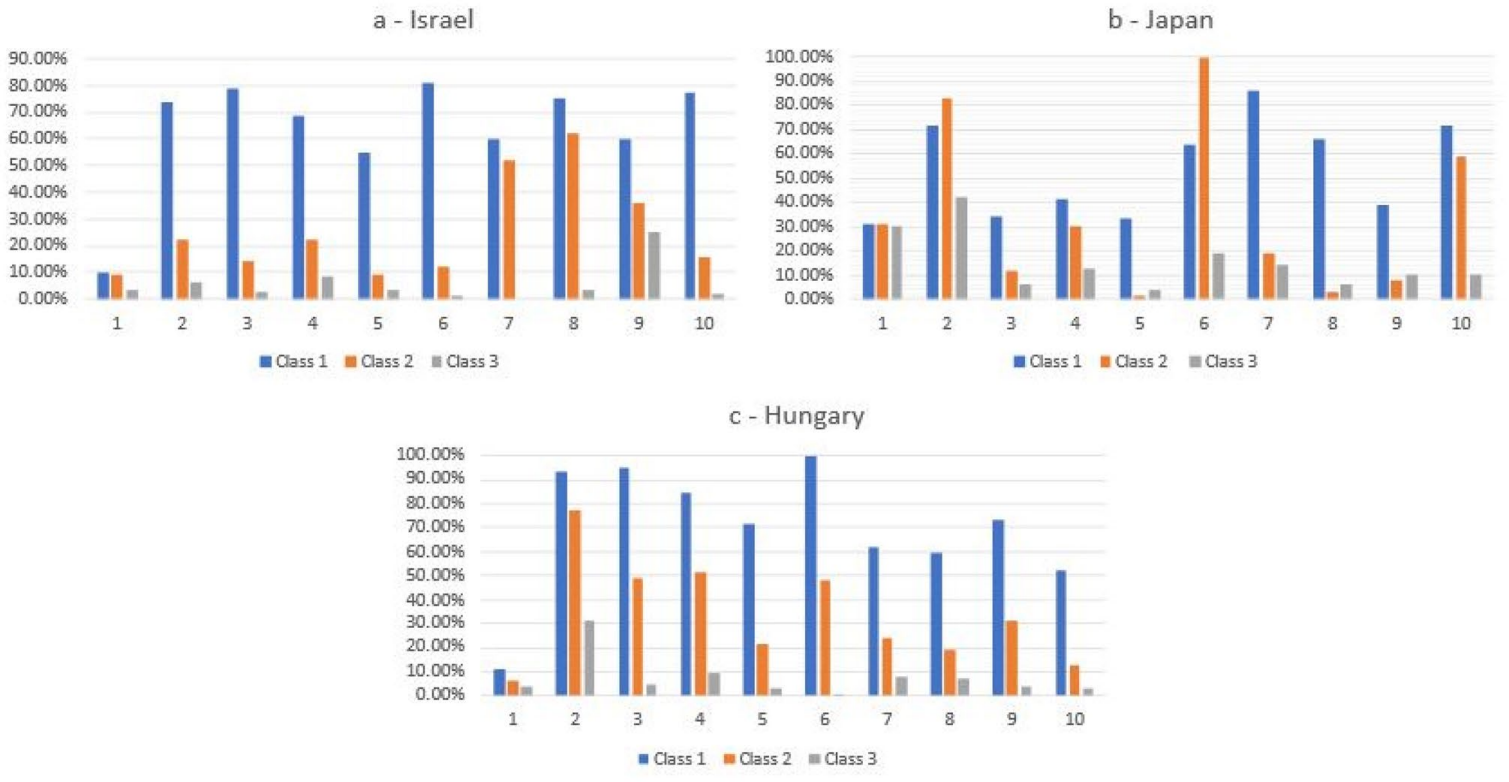
Three class solution of a Latent Profile Analysis of the False Belief scale. Items—1—The flu vaccine will protect you against COVID-19; 2—The COVID-19 vaccine causes allergies; 3—Vaccines weaken the immune system; 4—Vaccines do not cause autism (reverse coded, RC); 5—Vaccines do not contain mercury (RC); 6—The COVID-19 vaccine can give you covid-19; 7—If you have had COVID-19 already you can still benefit from the covid-19 vaccine (RC); 8—Receiving an mRNA vaccine will alter your DNA; 9—The vaccine has severe side-effects; 10—Reactions to the COVID vaccine are mild (RC).

A multi-group Latent Cluster Analysis tested one to four class solutions for the three samples, examining whether the solution demonstrated the same class pattern was obtained across samples^24^. As shown in Table 3, decrease in BIC was greatest for a three-class solution, providing strong evidence of best fit (in bold)^25^. Relative entropy for a three-class model indicated good classification accuracy (0.87 accuracy of class membership in any culture). Class-specific conditional probabilities for each indicator are displayed in Figs. 2a-c.

**Table 3:** Fit Indices for One-Four Multi-Group Latent Class Models.

	AIC	BIC	ssBIC	Entropy	(df) χ^2^
1-class	38,632.94	38,826.50	38,724.83	1.00	(2992) 17,427.94
2-class	35,454.51	35,835.58	35,635.40	.91	(3000) 6168.52
3-class	**34,925.15**	**35,493.73**	**35,195.06**	**.87**	**(2972) 4746.94**
4-class	34,679.88	35,435.97	35,038.80	.85	(2943) 3130.93

Abbreviations: AIC = Akaike Information Criterion, BIC = Bayesian Information Criterion, ssBIC = Sample Size Adjusted, Bayesian Information Criterion.

### Israel

We identified the three classes as **High False Beliefs** (n = 125, 12.3%) **vaccine may change DNA + overall Low False Beliefs** (n = 336, 33.2%) and **Low False Beliefs** (n = 550 54.4%). Separate Logistic Regression showed Classes 1 and 2 were associated with greater unwillingness to vaccinate (vs. the **low false belief** reference group, class 3). (χ^2^(1) = 215.97 p < 0.001). **High False Beliefs** was most closely associated with unwillingness to vaccinate (b = −3.36 SE = 0.26 Wald = 170.86 p < 0.001 OR = 0.035). **Vaccine may change DNA + Low False Beliefs** was also associated with unwillingness to vaccinate compared to the reference group (b = −1.14 SE = 0.18 Wald = 40.50, p < 0.001 OR 0.320).

### Hungary

We contrasted **High False Beliefs** (n = 136, 12.0%), **High belief in Allergies + overall moderate-Low False Beliefs** (n = 422, 37.3%) and **Low False Beliefs** (n = 573, 50.7%). Logistic Regression showed again that the first two classes were significantly associated with (un)willingness to vaccinate compared to the reference group (**low false beliefs**) (χ^2^(1) = 122.78 p < 0.001) (**High False Beliefs** (b = −2.84 SE = 0.43 Wald = 44.48 p < 0.001 OR = 0.058); **High belief in Allergies + overall moderate-Low False Beliefs** (b = −1.04 SE = 0.14 Wald = 51.79, p < 0.001 OR = 0.353).

### Japan

Classes were identified as **High-Moderate False Beliefs** (n = 115, 11.6%), **Low beliefs + Vaccine can give you COVID-19** (n = 427, 43.2%) and **Low False Beliefs** (n = 446, 45.1%). Separate Logistic Regression showed that the neither Class 1 nor Class 2 were associated with willingness to vaccinate, when compared to reference Class (3: **Low False Beliefs**): (χ^2^(1) = 1.08 p = 0.297: class 1 (b = −0.22 SE = 0.22 Wald = 0.99, p = 318 OR = 0.800); Class 2 (b = −0.09 SE = 0.14 Wald = 0.39, p = 531 OR = 0.915).

## Discussion

Across the world there is evidence of continued vaccine unease, with vaccine resistance identified as a top ten threat to global health even before COVID-19^26^. Countries however have fared differently in the availability of vaccines and the trust of their populations towards a growing range of possible vaccines, with a nested set of factors influencing uptake. In Israel, a well-established community-based health service, large-scale public health campaigns and the early purchase of a large number of vaccines helped the country achieve a rapid and comprehensive roll-out of the Pfizer-BioNtec vaccine^27,28^. Unsurprisingly therefore almost three-quarters of our sample in this country demonstrated willingness to be vaccinated. In contrast, our Hungarian and Japanese respondents were less willing to vaccinate. In our sample only just over half (51%) of Japanese respondents indicated such willingness, higher than the 45% reported by the Imperial College COVID-19 tracker in January 2021 but lower than the 62% indicated by a cross-sectional survey also conducted that month^11^. In Hungary, initial delays in accessing some vaccines, and the politicisation of the vaccine roll-out^7^, (including disputes over the use of vaccines not approved by the EU^8^), contributed to high national rates of scepticism about efficacy, with only 31% of our national sample expressing willingness to vaccinate, and a further 21% uncertain.

In competitive regressions demographic factors, and personal or family experience with COVID-19, had only a small association with vaccine willingness, although men were significantly more willing to vaccinate in Israel and Hungary. However, those who were more likely to regret not vaccinating were more likely to indicate vaccine willingness. There were only small and culturally variable associations between perceived likelihood or severity of infection and vaccine intention, indicating only a weak association between perceived threat and vaccination. This may be because while infection likelihood and severity are closely associated with viral threat, benefits and regrets may be more proximally associated with actual vaccine behaviour. Subjective pressure to vaccinate was significantly higher in Israel and Japan compared to Hungary and associated with vaccine uptake in just these first two countries. This suggests that the importance of friends, families and others for vaccine willingness may be particularly significant where important others are prepared to be vaccinated.

Trust in government emerged in all three countries as a significant contributor to vaccine willingness, as elsewhere^22^. This association was strongest in Hungary, where vaccine uptake, and choice of vaccine, has been particularly politically contentious. False information about the virus, most likely to emerge from social media, has been shown to be negatively associated with COVID-19 health protective behaviour^23^, including vaccine willingness^12^. In Israel, where we included also included religion, additional analyses found false beliefs to be strongest in the Ultra-Orthodox population, lowest in secular respondents (F (3, 1003) = 4.68 P = 0.003). As reported elsewhere^19,30^, misbeliefs were significantly associated with low trust in governmental authorities (rs −0.25,−0.24 and −0.32 for Israel, Japan and Hungary respectively, P = 0.001) but only the association in Israel between false beliefs and vaccine willingness survived the competitive regression models.

In our samples, misbeliefs about the vaccine correlated with each other, supporting the idea of a ‘monological belief system’^20,31^. However, in Israel the belief that *COVID-19 can alter your DNA* (held by 62% of those in the second category) was distinctive as a predictor of vaccine (un)willingness. Contrastingly, in Hungary it was the association between allergies and the vaccine (held by 77% of those in class 2) that distinguished a grouping of respondents reluctant to take the vaccine. In Japan, the largest latent class (43% of respondents) indicated their belief that *the vaccine can give you COVID-19.* Notably, 61.5% of Japanese respondents who believed *the vaccine can give you COVID* in Japan were unwilling to vaccinate, compared to 48.6% Israel and 14.0% in Hungary, suggesting the particular significance of this misbelief in Japan for vaccine intentions.

### Limitations

Our studies benefitted by including national samples from three very different cultures, with different histories of vaccine uptake. However, we recognise a number of limitations to our survey. First, samples were cross-sectional, and were therefore not able to assess predictors of vaccine willingness over time. Data was first collected in early January, at the start of the first major vaccine roll-out, meaning that we did not include later misbeliefs that emerged in subsequent months. Emergent concerns over vaccine safety (such as worries about blood clotting following the AstraZeneca vaccine^32^) may serve to directly inhibit uptake and perpetuate further new misbeliefs and distrust. Second, because of the speed of the evolving vaccination situation in both countries our survey companies expedited data collection within a short time period. Although widely used, and particularly particularly for the collection of time-sensitive data during a vaccination campaign, we recognise that the quota sampling has important limitations in ascertaining accurate response rates^12,33^. In Hungary in particular the low willingness to vaccinate may have been impacted by the omission in our data of those already vaccinated. Third, we assessed only three countries; future work should expand the testing of nested models across settings. We were not able to assess income or family structure across all countries, so did not include this in our analyses: both factors might be valuable further predictors of vaccine willingness. Finally, we measure only behavioural intentions rather than actual vaccination behaviour. Although the link between the two has been well established^34^ we recognise that attitudes towards any vaccination are likely to vary as populations acquire further experience with the vaccination rollout.

### Implications for vaccine drives

Despite these limitations we believe our findings have important implications as other nations strive to accelerate their vaccination drives. Vaccine campaigns may need not focus on disease threat: instead, these initiatives would better focus on the effectiveness of the vaccine, confront misinformation, and seek to emphasise the trustworthiness of key actors, such as national health services. Those vaccinated should be encouraged to inform close others, in order to emphasise the normative nature of this activity. Public health agencies need to reach people beyond remote media campaigns and be present where individuals shop and work^35^; doctors have been widely reported to be important in addressing myths^36^ with pharmacists in Hungary significant for encouraging influenza vaccination in that country^29^. Our analyses suggested that, despite some similarities in belief structure, there were distinctive beliefs in each culture important for understanding vaccine willingness. Finally, within country, group factors may be also particularly important, including culturally variable myths about COVID-19^37^. We note that in some countries (e.g. Israel), uptake has been greater in settlements with higher socio-economic status, despite the greater morbidity from COVID-19 amongst poorer communities^38^. To address these variations in uptake the specific concerns of religious and other social groups need to be considered, with community leaders actively engaged through culturally appropriate conversations in order to allay fears, address specific myths and thus help further facilitate a successful vaccine campaign^39^.

## Methods

### Participants and procedure

Data were from nationally representative samples of adult populations collected in Israel (N = 1011), Japan (N = 997) and Hungary (N = 1130), using large panel survey companies in each country (iPanel for Israel, Quesant! for Japan, Medián Opinion and Market Research in Hungary). Eighty respondents in Japan did not respond to the vaccination willingness item but provided other data (e.g. COVID-19 myths). In each country quota sampling was used to ensure the sample: participant sex, age (Israel), sex, age and geographical region (Japan, Hungary) were chosen to match population parameters in each country. Data in Israel were collected from 31.12.2020 to 11.01.2021 i.e. early on during the first vaccination drive. At that time the percentage of those receiving their first vaccination doubled, from 11 to 22% of the population^2^. In Hungary, data were obtained from 8.4.21 to 16.4.21, during which 27.8% (then 32.0%) had received at least one vaccine^2^. In Japan data were collected prior to the start of the vaccination campaign (between 15.2.2021 and 16.2.2021).

For each sample participants were contacted as part of cloud panels administered by the survey company and asked to participate via email. They were then reimbursed by the companies for their participation. All participants provided informed consent before proceeding with the study. In each country inclusion criteria required participants be the approved minimal age set by ethical requirements (Israel, Hungary—18, Japan—20), and to successfully pass validation checks (both specific items and timing of responses) to ensure participant attention and accuracy. All respondents were fluent in the relevant national language. Ethical approval was from Ariel University’s Institutional Review Board (No. AU-SOC-MBE-20201224), the Yamaguchi University Review Committee for Non-Medical Research Involving Human Participants (2020-004-01) and the Eötvös Loránd University ethical review panel (PPK KEB 2021/130-2). All data collection was performed in accordance with the Declaration of Helsinki and the relevant guidelines and regulations in each country.

### Measures

#### Demographics

Participants in each country indicated their age, sex and education. Because demographic information procedures vary across countries we obtained slight variations in each country, in common with other such cross-cultural comparisons^12^, while retaining the core ecosystems model variables in each country for comparative analysis. In Japan and Israel respondents identified whether they completed only high school prior to University or were currently a student/had graduated; in Hungary students indicated their level of schooling. Education was then recoded to academic vs. non-academic education. Table 1 provides descriptive statistics for each country.

#### Health conditions

Respondents indicated their risk group membership using the US CDC risk group memberships (e.g. hypertension, diabetes). Participants also indicated whether they had been formally diagnosed with COVID-19 (yes, no), whether someone from their social circle had been thus diagnosed (yes, no), and their self-rated health (from bad (1) to excellent (4)).

#### Vaccine acceptance

Participants were asked *Would you be willing to accept a vaccine approved safe and effective by the government?* (strongly disagree to strongly agree)). Because we questioned respondents at the start of actual vaccine campaigns, rather than about a hypothetical vaccine, and were cautious about both the translation of ‘uncertain/undecided’ and the use of intermediate responses in some cultural contexts^40^ we treat this as a binary response, in line with much previous cross-national vaccine research^22^,Responses were classified as 1 (‘completely agree’ or ‘somewhat agree’) vs. 0 (undecided, unwilling, very unwilling)^3,22^. We provide individual scores on the five-point scale by country in the Supplementary Materials (Supplementary Table S2).

#### Predictors of vaccine willingness

We included eight potential predictors of vaccine willingness drawn from three major theoretical perspectives previously used to assess vaccine uptake: the Health Belief Model (HBM), the Theory of Planned Behaviour (TPB) and the Theory of Protection Motivation (TPM)^13,14,34^, as well as subsequent work on the influence of false beliefs and trust in authorities on vaccine willingness. Full items and response categories are reported in the Online Supplementary File (Supplementary Table S1), scale alphas in Table 1.

#### Analytic procedure

Since samples differed on some important demographic characteristics, we tested if one-to-one propensity-score matching should be used between cultural groups and between the willing and unwilling to vaccinate^41–43^. To estimate the propensity score, a multinomial and logistic regression model was used to predict culture (three groups) and the willingness to vaccinate (willing/unwilling) with the following covariate variables: age, sex, education and subjective rated health, using the Matching package for R. After the propensity score was estimated, we examined the extent to which matching produced balance^44^. Results showed no difference between the matched (mean of willingness to vaccinate = 1.75 unwilling to vaccinate = 2.33, variance ratio = 1.15) and unmatched samples (mean of willingness to vaccinate = 1.75 unwilling to vaccinate = 2.34, variance ratio = 1.06) (see also Supplementary Table S3). Mean differences were 73.28 and 74.14 for matched and unmatched samples.. As we would need to limit our analyses by reducing sample size substantially in order to use matching samples we used instead residuals from regressions for the purpose of controlling for unwanted effects in multivariable datasets and to produce unbiased parameter estimates^45^. Residual variables were saved in the regression procedure, to "clean" the variables from the effects of the covariates. In order to calculate residual scores, we conducted a logistic regression predicting willingness to vaccinate by age, gender, education and subjective rated health, as well as the interaction between them. We report residuals that control for the variance related to covariates in Supplementary Table S4.

We utilized layered multi-group logistic regression analysis using MPlus 8.1^46^ to test the ecological model. Data were analysed using maximum likelihood estimation with robust standard errors (MLR) to handle non-normal distributions. We used multi-group analyses^47^ to test if paths from the covariates, demographic factors and health status, individual cognitions, normative pressures, trust in government, belief in COVID-19 myths and willingness to be vaccinated varied by culture. We included participant information on their own (or social network’s) positive COVID-19 diagnoses. Missing data due to nonresponse ranged from 0.9% to 4%.

To analyse the structure of misbeliefs about the vaccine we employed Multi-Group Latent Class Analysis (MLCA) to identify subgroups within the three samples (Israel, Japan and Hungary). We use binary indicators of false beliefs. We specified models with one to four classes and compared the models to determine the optimal number of classes. Models with lower values on the Akaike Information Criterion (AIC), Bayesian Information Criterion (BIC), and sample-size adjusted Bayesian Information Criterion (aBIC) were prioritized. Entropy values and average latent class probabilities indicated classification accuracy, with preference for models with entropy values and probabilities of correct class assignment closer to 1.00^48^. Once optimal number of classes was determined we computed sample percentages assigned to each class and conditional probabilities by class, with labels for latent classes based on patterns of probabilities for endorsing each false belief. Datasets are available in the Open Science Framework (OSF) repository: https://osf.io/dm587/.

## Supplementary Information

Supplementary Information.
