# Supplementary material for: Psychological factors underpinning vaccine willingness in Israel, Japan and Hungary
Source: Sci Rep. 2022 Jan 10;12:439. doi: 10.1038/s41598-021-03986-2 (PMC8748514; doi:10.1038/s41598-021-03986-2)
Supplement: Supplementary file 1 — Supplementary Information. [file 41598_2021_3986_MOESM1_ESM.docx]

Supplementary Materials

Psychological factors underpinning vaccine willingness in Israel, Japan and Hungary

Robin Goodwin, Menachem Ben-Ezra, Masahito Takahashi, Lan-Anh Nguyen Luu, Krisztina Borsfay, Mónika Kovács. Wai Kai Hou, Yaira Hamama-Raz, Yafit Levin.

Supplementary Table S1: Cognitive and belief items included in the questionnaire.

| Scale, Response options |
| --- |
| **Likelihood of infection ((1) not at all to (5) very much)** |
| My chance of getting COVID-19 in the next few months is great  I am worried about the likelihood of getting COVID-19 in the near future  Getting COVID-19 is currently a possibility for me |
| **Perceived severity of COVID-19 infection ((1) not at all to (5) very much)** |
| Complications from COVID-19 are serious,”  I will be very sick if I get COVID-19  I am afraid of getting COVID-19 |
| **Benefits of vaccine ((1) not at all to (5) very much)** |
| Vaccination is a good idea because I feel will be less worried about catching COVID-19  Vaccination decreases my chance of getting COVID-19 or its complications  If I get vaccinated, I will decrease the risk of spreading the disease to others |
| **Barriers to vaccine ((1) not at all to (5) very much)** |
| The side-effects of COVID-19 will interfere with my usual activities  I cannot be bothered to get a COVID flu vaccination |
| **Anticipated regret ((1) strongly disagree to (7) strongly agree)** |
| If I did not have a COVID vaccination, I would later wish I had |
| **Subjective norms ((1) strongly disagree to (7) strongly agree)** |
| People who are important to me would approve of me having the COVID-19 vaccination  My family would approve of me having the COVID-19 vaccination  My friends would approve of me having the COVID-19 vaccination  I feel under pressure to have a COVID-19 vaccination  People who are important to me influence my decision to have the COVID-19 vaccination |
| **Beliefs (1(no) 2 (yes)** (R) indicates reverse coding |
| The flu vaccine will protect you against COVID-19  The COVID-19 vaccine causes allergies  Vaccines weaken the immune system  Vaccines do not cause autism (R)  Vaccines do not contain mercury (R)  The COVID-19 vaccine can give you covid-19  If you have had COVID-19 already you can still benefit from the covid-19 vaccine (R)  Receiving an mRNA vaccine will alter your DNA  The vaccine has severe side effects  Reactions to the COVID vaccine are mild (R) |
| **Trust in government (1) not at all to (5) very much)** |
| I trust the Government in general  I trust Government to deal with COVID-19  I trust Government regarding vaccination |

**Table S2: Breakdown of "Strongly disagree" to "Strong agree" to vaccinate**

|  | Israel | Japan | Hungary |
| --- | --- | --- | --- |
| Strongly disagree | 47 (4.6%) | 57 (6.2%) | 347 (30.7%) |
| Disagree | 89 (8.8%) | 138 (15.0%) | 198 (17.5%) |
| Neutral | 125 (12.4%) | 253 (27.6%) | 235 (20.8%) |
| Agree | 329 (32.5%) | 312 (34.0%) | 168 (14.9%) |
| Strongly agree | 421 (41.6%) | 157 (17.1%) | 182 (16.1%) |

**Matching vs. no matching comparisons**

There were no significant differences in willingness to vaccinate between the cultural groups in unmatched patients and the matched samples. No significant differences were observed in the propensity score-matched participants (Table S3).

**Predicting willingness to vaccinate by residual scores**

Table S4 presents magnitude and statistical significance in a model predicting willingness to vaccinate adjusted for covariates (Residual Predictors) as well as adjusting the predictors for all covariates. This model represents the variables "cleaned" from the covariates presented in the main model. We note that regressing willingness to vaccinate on the residual variables can yield artificially high Betas and strong effects.

In **Israel**, we see positive associations between willingness to vaccinate and the cognitive factors of *benefits of vaccine* *and anticipated regret*, while *barriers to vaccination* were associated with reluctance to vaccinate. *Trust in government* was positively associated with willingness to vaccinate, and *false beliefs about COVID-19* were associated with unwillingness to vaccinate. In **Japan,** positive associations were identified between willingness to vaccinate and the cognitive factors of *anticipated regret* if not vaccinated and *benefits of vaccine*, while *barriers to vaccination* were associated with reluctance to vaccinate. Personally having experienced COVID-19 was also associated with less willingness to vaccinate, but family members having had the virus was associated with greater vaccine willingness. Higher *Likelihood of infection* and *lower perceived severity* of infection were associated with unwillingness to vaccinate. *Subjective norms* were associated with willingness to vaccinate as was *trust in government*. Finally, in **Hungary,** being in a risk group was associated with less vaccine willingness as was having family members who had been infected. There were positive associations between willingness to vaccinate and higher *benefits of vaccine* and *lower barriers to vaccine* and *trust in government*. The variances explained were 41.8%, 83.2% and 32.2% in Israel, Japan and Hungary, respectively.

**R script for propensity score matching with automated balance optimization**

library(haven)

Read <- read_sav("C:/Users/User/Desktop/Read.sav")

View (Read)

library("Matching")

data ("Read")

attach (Read)

**###Save the outcome of interest in Y and the treatment indicator**

Y <- Read$Cult

Tr <- Read$Will_n

**##We now estimate our first propensity score model:**

glm1 <- glm(Tr ~ Age + Educ + Sex + SRH, data = Read)

**##Let us do one-to-one matching with replacement using our preliminary propensity score model**

rr1 <-Match (Y = Y, Tr = Tr, X = glm1$fitted)

**# or just**

rr2<- MatchBalance(Tr ~ Cult, match.out = rr1, nboots = 1000, data = Read)

qqplot(Read$Will_n[rr1$index.control], Read$Will_n[rr1$index.treated])

abline(coef = c(0, 1), col = 2)

**Mplus Script for the main model**

DATA: FILE IS C:\Users\User\Desktop\Read.dat;

VARIABLE:

MISSING ARE ALL (-999);

NAMES ARE Sex Age Educ SRH Risk diag faDiag Will_n Regre perLike

perSev Benef Barri S_nor FB GOV Cult;

USEVARIABLES = Will_n Sex Age Educ SRH Risk diag faDiag Regre perLike

perSev Benef Barri S_nor FB GOV Cult;

classes is C (3);

KNOWNclass is C (Cult = 1 Cult = 2 Cult = 3);

ANALYSIS:

TYPE=MIXTURE;

ALGORITHM=INTEGRATION;

ESTIMATOR = MLR;

MODEL:

%OVERALL%

Will_n ON Sex Age Educ SRH Risk diag faDiag Regre perLike

perSev Benef Barri S_nor FB GOV;

%c#1%

Will_n ON Sex Age Educ SRH Risk diag faDiag Regre perLike

perSev Benef Barri S_nor FB GOV;

%c#2%

Will_n ON Sex Age Educ SRH Risk diag faDiag Regre perLike

perSev Benef Barri S_nor FB GOV;

%c#3%

Will_n ON Sex Age Educ SRH Risk diag faDiag Regre perLike

perSev Benef Barri S_nor FB GOV;

Output: sampstat standardized STDYX TECH1 TECH4 TECH8 TECH10 TECH14 cinterval(bootstrap);

**Table S3. Comparison between matched and unmatched samples (output from above script)**

Before Matching After Matching

mean vaccine........ 1.7459 1.7459

mean notvaccine..... 2.3308 2.3377

std mean diff......... -73.282 -74.144

mean raw eQQ diff..... 0.58572 0.62918

med raw eQQ diff..... 1 1

max raw eQQ diff..... 1 2

mean eCDF diff........ 0.19497 0.20973

med eCDF diff........ 0.27357 0.30828

max eCDF diff........ 0.31136 0.32091

var ratio (Tr/Co)..... 1.1491 1.0642

T-test p-value........ < 2.22e-16 < 2.22e-16

KS Bootstrap p-value.. < 2.22e-16 < 2.22e-16

KS Naive p-value...... < 2.22e-16 < 2.22e-16

KS Statistic.......... 0.31136 0.32091

Table S4. Predicting Willingness to Vaccinate Adjusted for covariates Residual Predictors - Magnitude, Statistical Significance

| Hungary (N = 1,130) | | | | Japan (N = 917) | | | | Israel (N = 1,011) | | | |  |
| --- | --- | --- | --- | --- | --- | --- | --- | --- | --- | --- | --- | --- |
| P | Est\Se | SE | b | P | Est\Se | SE | b | P | Est\Se | SE | b |  |
| .001 | -3.29 | .02 | **-.06**** | .057 | -1.91 | .01 | -.02 | .685 | -.41 | .02 | -.01 | Risk (1=risk group) |
| .337 | .96 | .00 | .00 | .046 | -.99 | .01 | **-.02*** | .731 | .34 | .03 | .01 | Had Covid (1=yes) |
| .027 | -2.21 | .02 | **-.04*** | <.001 | 4.21 | .01 | **.03***** | .928 | -.09 | .02 | -.00 | Family Covid (1=yes) |
| .641 | .47 | .02 | .01 | <.001 | -7.84 | .00 | **-.02***** | .073 | 1.79 | .01 | .02 | Perceived likelihood |
| .099 | 1.65 | .01 | .03 | <.001 | 7.89 | .00 | **.02***** | .823 | -.22 | .01 | -.00 | Perceived severity |
| .001 | 3.30 | .01 | **.04**** | <.001 | 54.13 | .00 | **.19***** | <.001 | 3.88 | .02 | **.05***** | Benefit of vaccine |
| <.001 | -9.85 | .00 | **-.10***** | <.001 | -57.01 | .01 | **-.19***** | <.001 | -10.06 | .01 | **-.11***** | Barriers to vaccine |
| .570 | -.57 | .01 | .00 | <.001 | 3.74 | .01 | **.03***** | <.001 | 8.39 | .01 | **.07***** | Anticipated Regret |
| .340 | -.95 | .01 | -.01 | <.001 | 3.73 | .01 | **.03***** | .086 | 1.72 | .01 | .02** | Subjective Norms |
| <.001 | 9.89 | .01 | **.11***** | .021 | 2.31 | .01 | **.01*** | .052 | 1.95 | .01 | **.02*** | Trust in Government |
| .156 | -1.42 | .06 | -.09 | .791 | -.27 | .00 | .00 | <.001 | -5.47 | .07 | **-.36***** | False Beliefs |

*Note:* ^***^p<.001; ^**^p<.01 ^*^p<.05
